# Changes in blood pressure and arterial stiffness monitored using the cardio–ankle vascular index during hemodialysis

**DOI:** 10.3389/fphys.2023.1133037

**Published:** 2023-02-20

**Authors:** Shuji Sato, Kazuhiro Shimizu, Mao Takahashi, Motoyuki Masai, Osamu Nagakawa, Junji Uchino, Toshihiro Suzuki, Yuka Sato, Noriko Iwai, Kohji Shirai

**Affiliations:** ^1^ Department of Cardiology, Toho University Sakura Medical Center, Chiba, Japan; ^2^ Mihama Hospital, Chiba, Japan

**Keywords:** CAVI, arterial stiffness, hemodialysis, intra-dialytic hypotension, vascular function

## Abstract

During hemodialysis (HD), blood pressure (BP) changes are frequently observed. However, the mechanism of BP changes during HD has not been fully clarified. The cardio–ankle vascular index (CAVI) reflects the arterial stiffness of the arterial tree from the origin of the aorta to the ankle independent from BP during measurement. Additionally, CAVI reflects functional stiffness in addition to structural stiffness. We aimed to clarify the role of CAVI in regulating the BP system during HD. We included 10 patients undergoing 4-hour HD (total 57 HD sessions). Changes in the CAVI and various hemodynamic parameters were evaluated during each session. During HD, BP decreased and CAVI significantly increased (CAVI, median [interquartile range]; 9.1 [8.4–9.8] [0 min] to 9.6 [9.2–10.2] [240 min], *p* < 0.05). Changes in CAVI from 0 min to 240 min were significantly correlated with water removal rate (WRR) (r = −0.42, *p* = 0.002). Changes in CAVI at each measurement point were negatively correlated with ΔBP (Δsystolic BP_each MP_, r = −0.23, *p* < 0.0001; Δdiastolic BP_each MP_, r = −0.12, *p* = 0.029). Whereas one patient exhibited a simultaneous decrease in BP and CAVI during the initial 60 min of HD. Arterial stiffness monitored with CAVI generally increased during HD. CAVI elevation is associated with decreased WWR and BP. An increase in CAVI during HD may reflect the contraction of smooth muscle cells and play an important role in BP maintenance. Hence, measuring CAVI during HD may distinguish the cause of BP changes.

## 1 Introduction

Blood pressure (BP) changes can occur during hemodialysis (HD) and hypotension attack and is associated with severe clinical symptoms such as fainting, discomfort, and sweating. Intra-dialytic hypotension occurs in 10%–12% of HD cases and is related to poor prognosis (Gul et al., 2016; Sars et al., 2020). The changes in BP during HD is thought to be mainly caused by the removal of the volume of the water component. Other factors involved in BP changes are the autonomic nervous system and cardiac function (Sars et al., 2020). In addition, the contraction of arterial smooth muscle cells may be involved in controlling BP, but understanding the state of arterial smooth muscle contraction or relaxation is difficult.

Arterial stiffness consists of structural and functional stiffness. The former mainly reflects arteriosclerosis and vascular aging. The latter reflects arterial smooth muscle contraction (Lacolley et al., 2017). As an index of arterial stiffness, pulse wave velocity (PWV) has been used to mainly evaluate the degree of arteriosclerosis or vascular aging, and there were several reports that PWV played a predictive role in cardiovascular events (Blacher et al., 1999; Vlachopoulos et al., 2010). As for changes in PWV during HD, Lafta et al. (2022) demonstrated that there were no significant changes in PWV during HD. Öğünç et al. (2015) reported that BP and PWV decreased during HD. However, PWV is dependent on BP during measurement (Cecelja and Chowienczyk, 2009; Schillaci et al., 2015). Therefore, PWV is not an appropriate index for the evaluation of arterial stiffness if BP changes during HD.

Recently, the cardio–ankle vascular index (CAVI) was developed as a BP-independent arterial stiffness index by applying the stiffness parameter *ß* theory (Shirai et al., 2006). The CAVI reflects arterial stiffness from the origin of the aorta to the ankle, and arterial stiffness indicated by CAVI is composed of structural stiffness, which reflects the degree of arteriosclerosis (Shirai et al., 2011a), and functional stiffness (Shirai et al., 2011b). Functional stiffness reflects contraction or relaxation of arterial smooth muscle cells. Thus, CAVI may be a useful index for evaluating the role of arterial smooth muscle contraction in various pathological conditions. Nagasawa et al. (2022) reported that CAVI increased in hypovolemic states caused by acute blood removal in rabbits. This result strongly suggests that CAVI could reflect systemic vascular contraction in hypovolemic states. This hypovolemic state resembles the hemodynamic condition during HD. Therefore, measuring CAVI during HD may provide insight into understanding vascular conditions and may lead to clarify the pathophysiological mechanisms of the BP change during HD. CAVI could be a novel modality for assessing vascular physiology during HD.

In this study, we measured CAVI during HD to clarify the role of CAVI in maintaining systemic circulation.

## 2 Materials and methods

We enrolled 10 patients who underwent a 4-hour HD therapy at Mihama Hospital between March 2015 and May 2015 for a total of 57 HD sessions (6 HD sessions per patient, 1 patient had 3 HD sessions). Patients with persistent atrial fibrillation, peripheral arterial disease with ankle–brachial index <0.9, and severe aortic valvular heart disease were excluded from this study.

We analyzed the systolic BP (SBP), diastolic BP (DBP), heart rate (HR), heart–ankle PWV (haPWV), CAVI, and stroke volume (SV) during HD. The BP, HR, haPWV, and CAVI were measured using VaSela VS-1500 (Fukuda Denshi Co. Ltd., Tokyo, Japan) after 10 min of rest in the supine position. Simultaneously, SV was non-invasively measured using an impedance cardiac output meter (AESCULON; Osypka Medical Inc., Berlin, Germany). Systemic vascular resistance (SVR) was calculated as the ratio of mean BP to cardiac output (CO).

To evaluate the intra-dialytic changes in arterial stiffness and hemodynamics, each parameter was measured at the following points during each HD session: before HD (0 min), 30 min, 90 min, 150, 210, and 240 min, and after blood return. To examine the relationship between changes in CAVI or various hemodynamic parameters and water removal volume (WRV), the correlation between changes in CAVI or other parameters from 0 min to 240 min (ΔCAVI_0–240 min_, ΔSBP_0–240 min_, ΔDBP_0–240 min_, ΔHR_0–240 min_, ΔSV_0–240 min_, ΔCO_0–240 min_, and ΔSVR_0–240 min_) and water removal rate (WRR), which was defined as the percentage of WRV per body weight before HD, were analyzed. To examine the relationship between CAVI and other parameters at each measurement point (MP), the correlation between the changes in CAVI at each MP (ΔCAVI_each MP_) and the changes in other parameters at the same time (ΔSBP_each MP_, ΔDBP_each MP_, ΔHR_each MP_, ΔSV_each MP_, ΔCO_each MP_, and ΔSVR_each MP_) were also analyzed.

Furthermore, CAVI was measured every 30 min in one patient whose BP decreased in the initial 60 min during HD.

### 2.1 Statistical analyses

Data normality was assessed using the Shapiro–Wilk test. For patient characteristics, continuous variables are expressed as mean ± standard deviation or median (interquartile range [IQR]), as appropriate. Categorical variables are expressed as percentages. Hemodynamic parameters, including CAVI, are expressed as median (IQR). Comparisons of hemodynamic parameters during HD were performed using the Friedman test and the *post hoc* Steel–Dwass test. We analyzed the association between changes in CAVI and other hemodynamic parameters using Spearman’s correlation coefficient. The statistical significance was set at a *p* < 0.05. Statistical analysis was performed using EZR on R commander statistical software version 1.55 (Saitama Medical Center, Jichi Medical University, Saitama, Japan) (Kanda, 2013) and JMP computer software version 14.2 (SAS Institute, Cary, NC, United States).

### 2.2 Ethics approval

Informed consent was obtained from all patients. This study was approved by the ethics committee of Toho University Sakura Medical Center (S18019) and Mihama Hospital (17-010) and was performed in accordance with the latest version of the tenets of the Declaration of Helsinki.

## 3 Results

### 3.1 Patient characteristics

A total of 10 patients undergoing HD were enrolled in this study. All patients received 4-hour HD for 3 times per week; 9 patients who had 6 HD sessions and 1 patient who had 3 HD sessions were enrolled in the study, thus providing a total of 57 HD sessions for analysis.

Table 1 summarizes patient characteristics. The study sample comprised 50% men, and the mean age was 69.3 ± 9.3 years. The median dialysis duration was 4.7 years. The percentage of patients with diabetes mellitus was 20%, and the percentage of those with a history of coronary artery disease was also 20%. Echocardiographic findings revealed a mean left ventricular ejection fraction of 64.0% ± 6.7%. The mean E/e' was 17.5 ± 4.3, thus indicating left ventricular diastolic dysfunction. The rate of use of oral antihypertensive agents and hypoglycemic agents was low (Ca channel blocker, 10%; angiotensin Ⅱ receptor blocker, 20%; *ß*-blocker, 30%; and hypoglycemic agent, 10%). Mean total WRV per single HD session was 2992.6 ± 564.2 mL, and mean WRR was 5.5% ± 1.3%.

**TABLE 1:** Patient characteristics.

	Hemodialysis patients (*N* = 10)
Male n (%)	5 (50)
Age (years)	69.3 ± 9.3
BMI (kg/m^2^)	21.5 ± 3.2
Duration of hemodialysis (years)	4.7 (2.2–6.8)
DM (%)	2 (20)
HT (%)	4 (40)
DL (%)	1 (10)
CAD (%)	2 (20)
Etiology of renal failure	
Chronic nephritis (%)	4 (40)
Diabetic nephropathy (%)	2 (20)
Renal sclerosis (%)	2 (20)
Unknown (%)	2 (20)
Cardiac function	
LVEF (%)	64.0 ± 6.7
E/e'	17.5 ± 4.3
Medications	
CCB (%)	1 (10)
ARB (%)	2 (20)
*ß*-blocker (%)	3 (30)
Statin (%)	1 (10)
Insulin (%)	1 (10)
Hypoglycemic agent (%)	1 (10)
Nitrate (%)	2 (20)
Nicorandil (%)	1 (10)
Total WRV (mL) (per single hemodialysis session)	2992.6 ± 564.2
WRR (%) (per single hemodialysis session)	5.5 ± 1.3

Data are shown as mean ± standard deviation or median (interquartile range) or number (%). BMI, body mass index; DM, diabetes mellitus; HT, hypertension; DL, dyslipidemia; CAD, coronary artery disease; LVEF, left ventricular ejection fraction; E, peak early diastolic transmitral flow velocity; e’, peak early diastolic annular velocity; E/e’, ratio of E to e’; CCB, calcium channel blocker; ARB, angiotensin II, receptor blocker; WRV, water removal volume; WRR, water removal rate.

### 3.2 Changes in blood pressure, cardio–ankle vascular index, and other hemodynamic parameters during hemodialysis

**FIGURE 1:**
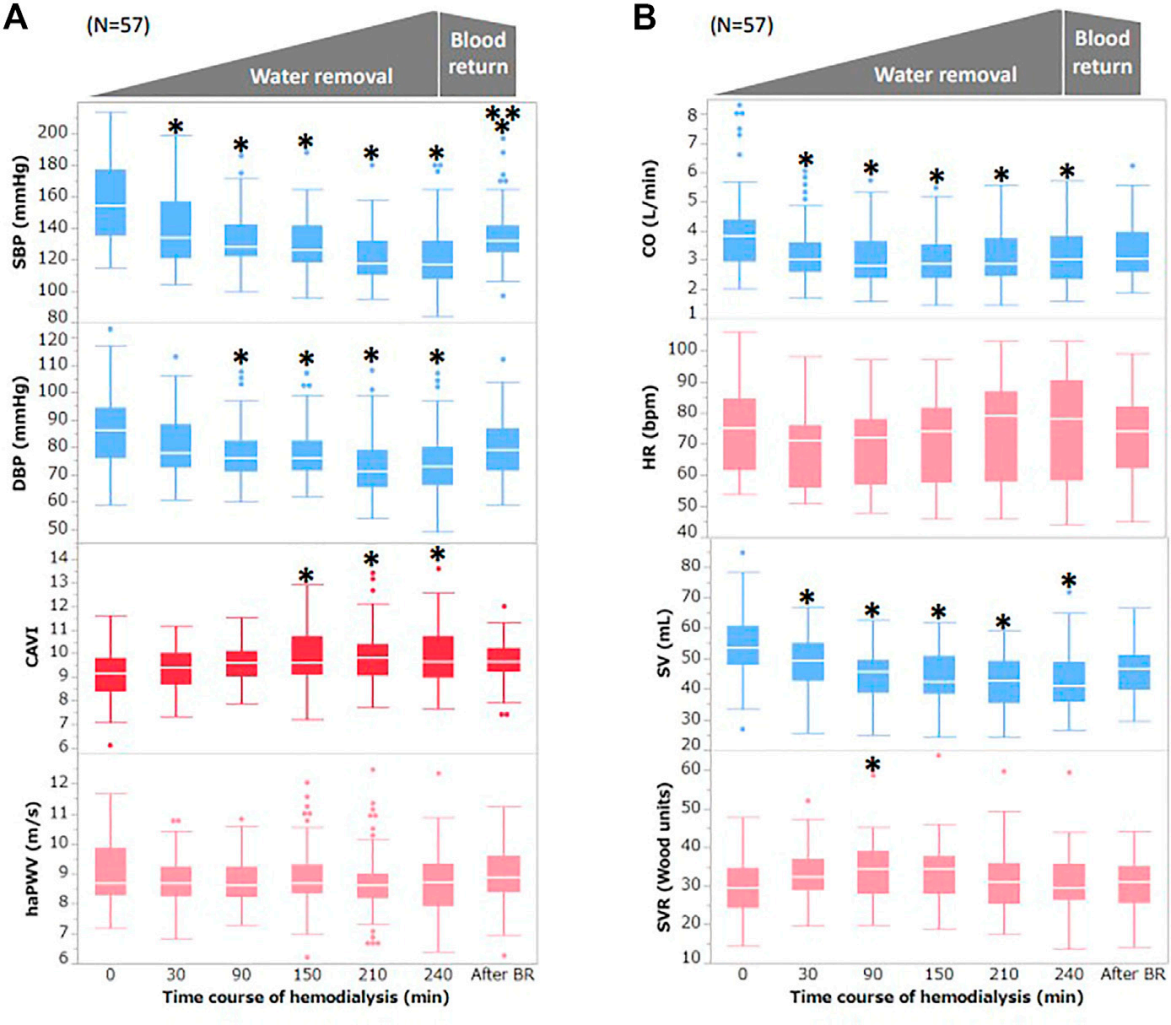
Changes in BP, CAVI, and other hemodynamic parameters during hemodialysis **(A)** Changes in BP, CAVI, and haPWV during hemodialysis. **(B)** Changes in CO, HR, SV, and SVR during hemodialysis. Data are shown as median (interquartile range). White bars, median; boxes, interquartile range; whisker, ranges excluding statistical outliers; plots, statistical outliers. SBP, systolic blood pressure; DBP, diastolic blood pressure; CAVI, cardio–ankle vascular index; haPWV, heart–ankle pulse wave velocity; CO, cardiac output; HR, heart rate; SV, stroke volume; SVR, systemic vascular resistance. *, *p* < 0.05 vs. 0 min; **, *p* < 0.05 vs. 240 min.

Figure 1A depicts the changes in BP, CAVI, and haPWV during HD. The SBP significantly decreased from 30 min to 240 min and after blood return. The DBP significantly decreased from 90 min to 240 min. After blood return, both SBP and DBP increased compared with their values at 240 min. In contrast to the BP, CAVI significantly increased from 150 min to 240 min. For haPWV, there were no significant changes during HD or after blood return.

Figure 1B depicts the changes in CO, HR, SV, and SVR during HD. CO significantly decreased from 30 min to 240 min. HR did not change significantly during HD but increased slightly from 210 min to 240 min. SV significantly decreased from 90 min to 240 min and after blood return. After blood return, SV tended to increase compared with that at 240 min but was not statistically significant. SVR significantly increased at 90 min, but there was no significant change at other MPs.

### 3.3 Correlation between changes in cardio–ankle vascular index or blood pressure and water removal rate during hemodialysis

**FIGURE 2:**
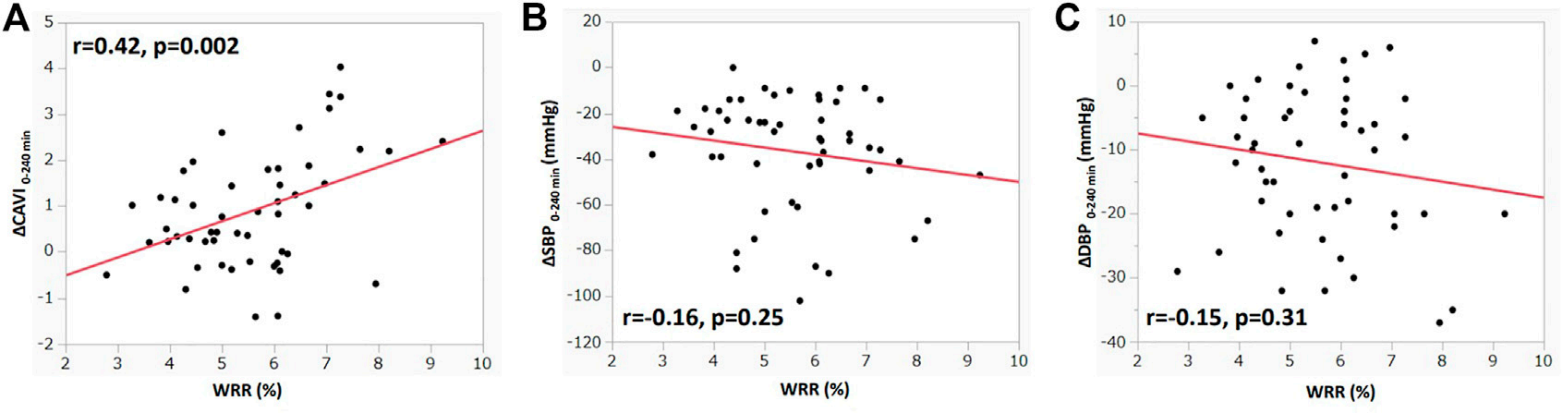
Correlation between ΔCAVI_0–240 min_ or ΔBP_0–240 min_, and WRR during hemodialysis **(A)** Correlation between ΔCAVI _0–240 min_ and WRR during hemodialysis. **(B)** Correlation between ΔSBP_0–240 min_ and WRR during hemodialysis. **(C)** Correlation between ΔDBP_0–240 min_ and WRR during hemodialysis. r, Spearman’s correlation coefficient; CAVI, cardio–ankle vascular index; BP, blood pressure; WRR, water removal rate; SBP, systolic blood pressure; DBP, diastolic blood pressure.

Figure 2 shows the correlation between ΔCAVI_0–240 min_ or ΔBP_0–240 min_ and WRR. ΔCAVI_0–240 min_ was significantly correlated with WRR (r = 0.42, *p* = 0.002; Figure 2A). ΔSBP_0–240 min_ and ΔDBP_0–240 min_ tended to correlate with WRR, but the correlation was not statistically significant (ΔSBP_0–240 min_: r = −0.16, *p* = 0.25; ΔDBP_0–240 min_: r = −0.15, *p* = 0.31 vs. WWR; Figures 2B,C). ΔHR_0–240 min_, ΔSV_0–240 min_, ΔCO_0–240 min_, and ΔSVR_0–240 min_ were not significantly correlated with WRR (ΔHR_0–240 min_: r = 0.22, *p* = 0.12; ΔSV_0–240 min_: r = 0.10, *p* = 0.48; ΔCO_0–240 min_: r = 0.17, *p* = 0.22; ΔSVR_0–240 min_: r = −0.17, *p* = 0.21 vs. WWR).

### 3.4 Correlation between changes in cardio–ankle vascular index and blood pressure at each measurement point during hemodialysis

**FIGURE 3:**
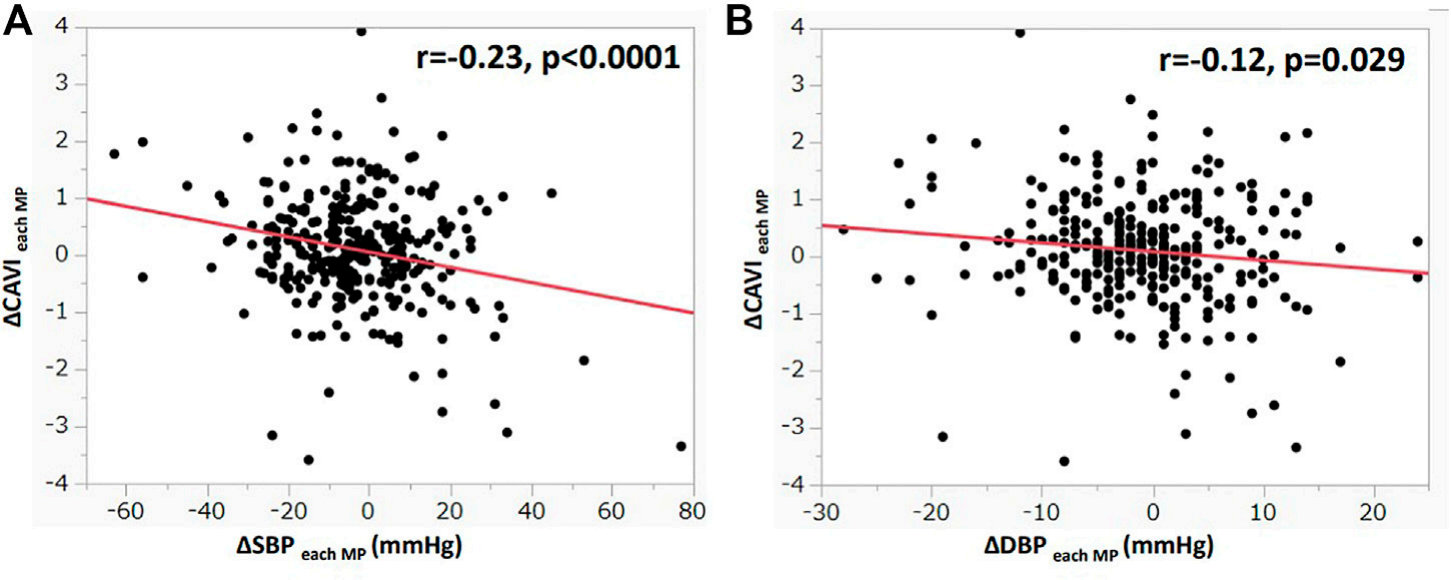
Correlation between ΔCAVI and ΔBP at each MP during hemodialysis **(A)** Correlation between ΔCAVI_each MP_ and ΔSBP_each MP_ during hemodialysis. **(B)** Correlation between ΔCAVI_each MP_ and ΔDBP_each MP_ during hemodialysis. r, Spearman’s correlation coefficient; CAVI, cardio–ankle vascular index; MP, measurement point; BP, blood pressure; SBP, systolic blood pressure; DBP, diastolic blood pressure.

Figure 3 shows the correlation between ΔCAVI and ΔSBP or ΔDBP at each MP during HD. ΔCAVI_each MP_ significantly correlated with ΔSBP_each MP_ and ΔDBP_each MP_ (ΔSBP_each MP_: r = −0.23, *p* < 0.0001; ΔDBP_each MP_: r = −0.12, *p* = 0.029; Figures 3A,B). In other parameters, ΔSV_each MP_ and ΔCO_each MP_ did not correlate with ΔCAVI_each MP_ (ΔSV_each MP_: r = 0.08, *p* = 0.15; ΔCO_each MP_: r = −0.008, *p* = 0.88). ΔCAVI_each MP_ was positively correlated with ΔHR_each MP_ and was negatively correlated with ΔSVR_each MP_ (ΔHR_each MP_: r = 0.21, *p* = 0.001; ΔSVR_each MP_: r = −0.14, *p* = 0.01).

### 3.5 A case with decreased blood pressure and cardio–ankle vascular index

**FIGURE 4:**
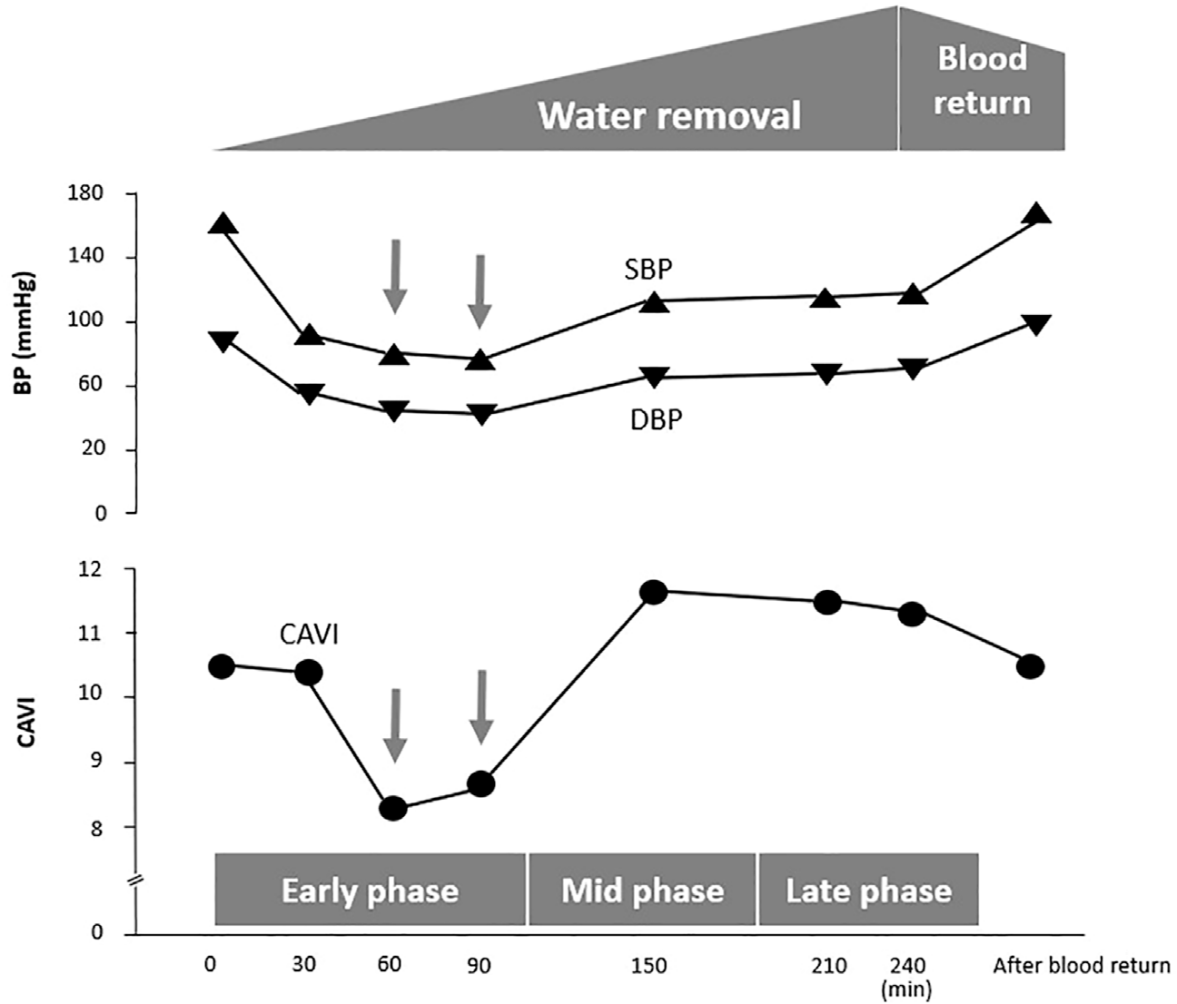
A case in which CAVI decreases with a reduction in BP at the early phase of hemodialysis. Case. An 85-year-old man with diabetic nephropathy. CAVI, cardio–ankle vascular index; SBP, systolic blood pressure; DBP, diastolic blood pressure.

An 85-year-old man exhibited a decrease in BP at 60 min during HD, as shown in Figure 4. At that moment, CAVI also decreased accompanying with the decrease in BP.

## 4 Discussion

In present study, we observed changes in hemodynamic parameters and arterial stiffness monitored with CAVI during HD, and the relationship between hemodynamic parameters and CAVI was evaluated. The SBP and DBP started to decrease at 30 min; this decrease continued until 240 min and then increased slightly after blood return, as shown in Figure 1A. A decrease in CO is also shown in Figure 1B. The observed decrease in these parameters was due to the decrease in circulating blood volume by water removal with HD. The WRR was negatively related to the changes in SBP and DBP but not significantly (SBP: r = −0.16, *p* = 0.25; DBP: r = 0.15, *p* = 0.31; Figures 2B,C).

Conversely, CAVI gradually increased from 30 min, and the increase was significant at 150 min to 240 min. The contradictory changes between BP and CAVI were also confirmed by significant correlations between ΔSBP and ΔDBP and ΔCAVI at each MP, as shown in Figure 3.

Reportedly, CAVI reflects functional stiffness in addition to structural stiffness of the arterial tree (Shirai et al., 2011b; Takahashi et al., 2012; Chiba et al., 2015; Shimizu et al., 2016; Sato et al., 2020). The adrenaline receptor α blocker doxazosin, which dilates arterial smooth muscles cells, is reported to decrease BP and decrease CAVI (Shirai et al., 2011b). Nitroglycerine, which also dilates smooth muscle cells, decreases CAVI (Chiba et al., 2015; Shimizu et al., 2016). Nagasawa et al. (2022) reported that blood removal provokes a decrease in BP and an increase in CAVI in rabbits. Moreover, in this experimental study, actual vasoconstriction was observed using intra-vascular ultrasound. This fact strongly suggests that the CAVI increase reflects vasoconstriction during blood removal. These results indicate that CAVI reflects the state of contraction and/or dilatation of smooth muscle cells in the arterial tree and that CAVI may be involved in the control system of BP as a part of resistance. From this viewpoint, an increase in CAVI during HD reflects smooth muscle contraction in the arterial tree from the origin of the aorta to the ankle and increases BP as a part of systemic resistance. The mechanism by which CAVI increases during blood removal may be *via* the stimulation of the sympathetic autonomic nervous system by hypotension. However, more studies are required to understand this further.

**FIGURE 5:**
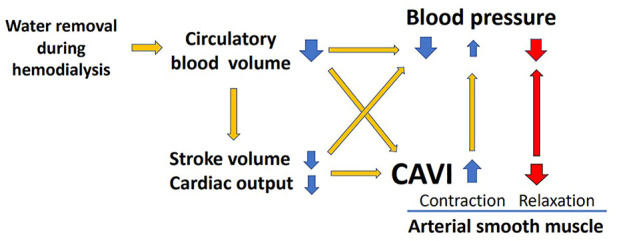
Role of CAVI in hemodynamic maintenance during hemodialysis. CAVI, cardio–ankle vascular index.

A weak negative relationship between WRR and BP (Figures 2B,C) was observed, but it was not statistically significant. The lack of significance in the relationship between WRR and BP may be owing to the compensatory reaction of CAVI; that is, an increase in CAVI by blood removal may have increased the peripheral arterial resistance and prevented a decrease in BP because the relationship between WRR and ΔCAVI was strongly positive (Figure 2A). From these results and speculations, we suggest that CAVI may play an important role in regulating systemic BP *via* peripheral artery resistance (Figure 5).

Furthermore, haPWV did not change during HD, as shown in Figure 1A. PWV essentially depends on BP upon measurement. Enhancing arterial stiffness brought about by water loss during HD should enhance PWV, but a decrease in BP reduced PWV, which depended on BP at measurement. Therefore, haPWV seemed to be unchanged. Furthermore, PWV is not a suitable index for reflecting proper arterial stiffness.

The above results suggested that an increase in CAVI during HD reflects the contraction of smooth muscle cells. This increase in CAVI might contribute to BP maintenance, which decreased during HD by decreasing the circulating blood brought about by water removal (Figure 5).

An 85-year-old man exhibited a decrease in BP at 60 min of HD, as shown in Figure 4. At this time, CAVI also decreased with the decrease in BP. This phenomenon demonstrates that smooth muscle cells dilate when BP decreases (Figure 5). The precise mechanism for smooth muscle dilation is not yet clear. However, those observations are important, because CAVI can differentiate the causes of a decrease in BP during HD. One general cause of a decrease in BP accompanying the elevation in CAVI is due to blood volume loss. The other cause of a decrease in BP accompanying a decrease in CAVI is due to smooth muscle cell dilatation or relaxation. Thus, the cause of a decrease in BP during HD might be identified by measuring CAVI.

In summary, arterial stiffness monitored with CAVI generally increased during HD. In addition, this CAVI increase was associated with an increase in WWR and a decrease in BP. An increase in CAVI during HD may reflect the contraction of smooth muscle cells and plays an important role in maintaining BP. On the basis of this hypothesis, measuring CAVI during HD may help to clarify the cause of BP change.

## 5 Study limitations

First, this was an observational study with a small sample size. We included only 10 HD patients that was not enough size for statistical analysis. Therefore, we analyzed total 57 HD sessions (6 sessions per patient except 1 patient) that were measured CAVI at same time points. Second, although the mechanism of increased CAVI was presumed to be due to the activation of the sympathetic nervous system, the precise mechanism has not been verified. Third, the number of cases in which CAVI decreased during HD was small, and we were not able to show mechanisms for the decrease in CAVI. Therefore, further studies with a large number of patients and on precise mechanism are needed to clarify.

## Data Availability

The raw data supporting the conclusion of this article will be made available by the authors, without undue reservation.
